# Assessment of *Staphylococcus Aureus* growth on biocompatible 3D printed materials

**DOI:** 10.1186/s41205-023-00195-7

**Published:** 2023-11-02

**Authors:** Nicole Senderovich, Sharan Shah, Thomas J. Ow, Stephanie Rand, Joshua Nosanchuk, Nicole Wake

**Affiliations:** 1 Albert Einstein College of Medicine, Montefiore Health System, Bronx, NY USA; 2 Department of Otorhinolaryngology – Head and Neck Surgery, Montefiore Health System, Albert Einstein College of Medicine, Bronx, NY USA; 3 Department of Pathology, Montefiore Health System, Albert Einstein College of Medicine, Bronx, NY USA; 4 Department of Physical Medicine & Rehabilitation, Montefiore Health System, Albert Einstein College of Medicine, Bronx, NY USA; 5 Department of Infectious Disease, Montefiore Health System, Albert Einstein College of Medicine, Bronx, NY USA; 6 Department of Research and Scientific Affairs, GE HealthCare, New York, NY USA; 7 Center for Advanced Imaging Innovation and Research (CAI²R) and Bernard and Irene, Schwartz Center for Biomedical Imaging, Department of Radiology, NYU Langone Health, NYU Grossman School of Medicine, New York, NY USA

**Keywords:** 3D printing, Laryngectomy tubes, Biofilm, Biocompatibility, Implantable device

## Abstract

The customizability of 3D printing allows for the manufacturing of personalized medical devices such as laryngectomy tubes, but it is vital to establish the biocompatibility of printing materials to ensure that they are safe and durable. The goal of this study was to assess the presence of *S. aureus* biofilms on a variety of 3D printed materials (two surgical guide resins, a photopolymer, an elastomer, and a thermoplastic elastomer filament) as compared to standard, commercially available laryngectomy tubes.

C-shaped discs (15 mm in height, 20 mm in diameter, and 3 mm in thickness) were printed with five different biocompatible 3D printing materials and *S. aureus* growth was compared to Shiley™ laryngectomy tubes made from polyvinyl chloride. Discs of each material were inoculated with *S. aureus* cultures and incubated overnight. All materials were then removed from solution, washed in phosphate-buffered saline to remove planktonic bacteria, and sonicated to detach biofilms. Some solution from each disc was plated and colony-forming units were manually counted the following day. The resulting data was analyzed using a Kruskal-Wallis and Wilcoxon Rank Sum test to determine pairwise significance between the laryngectomy tube material and the 3D printed materials.

The Shiley™ tube grew a median of 320 colonies (IQR 140–520), one surgical guide resin grew a median of 640 colonies (IQR 356–920), the photopolymer grew a median of 340 colonies (IQR 95.5–739), the other surgical guide resin grew a median of 431 colonies (IQR 266.5–735), the thermoplastic elastomer filament grew a median of 188 colonies (IQR 113.5–335), and the elastomer grew a median of 478 colonies (IQR 271–630). Using the Wilcoxon Rank Sum test, manual quantification showed a significant difference between biofilm formation only between the Shiley™ tube and a surgical guide resin (p = 0.018).

This preliminary study demonstrates that bacterial colonization was comparable among most 3D printed materials as compared to the conventionally manufactured device. Continuation of this work with increased replicates will be necessary to determine which 3D printing materials optimally resist biofilm formation.

## Introduction

Three-dimensional (3D) printing is an innovative technology, the applications of which have the potential to significantly improve patient outcomes. 3D printing allows for the manufacturing of complex anatomical structures and is already used for surgical training, pre-surgical planning, prosthetics, and implants in a variety of medical fields [1, 2]. This technology has been utilized in many clinical scenarios including modeling complex renal and prostatic cancers, orthopedic injuries, as well as congenital heart disease; and it has allowed for improved pre-surgical planning and can positively impact surgical outcomes [3–7]. 3D printing has also been used by original equipment manufacturers for the production of medical devices [8].

Materials used for the printing of implantable devices include polymers such as polyethylene, biological materials such as collagen and cellulose, ceramics, and metals such as titanium and cobalt chrome. Biodegradable vascular stents and prosthetic heart valves have been manufactured using 3D printing from polylactic acid (PLA), polycaprolactone (PCL) powders and methacrylate composite hydrogels, respectively [9]. Furthermore, 3D printing has been used to create titanium orthopedic implants [10].

In the field of otorhinolaryngology, 3D printed devices made from PCL have been used to replace airways in newborns and infants with tracheobronchomalacia [11, 12]. 3D printed, customized, silicone implants have been used in rhinoplasty without complication [13]. All of these devices carry significant potential advantages, including personalization of these items, as well as improved availability due to point-of-care acquisition. Investigators have also been able to use 3D printing to create reliable structures for maxillofacial reconstruction using thermoplastics and light-cured resins. These models have yet to be used in patients but were shown to be incredibly precise and serve as a springboard for future studies [14]. The customizability of 3D printed technology allows for personalized devices that can be well-tolerated by patients. Furthermore, the ease of accessibility to 3D printed devices when made in-house, as well as their cost-effectiveness, can allow for quicker and more practical solutions for patient needs.

Silicone is often preferred for soft-tissue implants as it is less susceptible to biofilm formation than materials such as acrylic and polymethylmethacrylate (PMMA) [15]. However, titanium is more commonly used for bony implants due to its mechanical strength and fatigue resistance [9]. The use of biocompatible 3D printed resins raises an important concern: the increased porosity and surface irregularity caused by 3D printing introduces the possibility of more biofilm formation [16]. Due to the potential for 3D printing to provide customizable patient implants, it is important that 3D printed devices created at the point of care are subject to the same microbial testing as traditionally manufactured devices that have 510(k) clearance to be marketed as safe and effective.

All medical devices deemed “biocompatible” must comply with the ISO 10993 standards. This set of standards assesses device safety by examining several key components of material usage including: contact duration, cytotoxicity, sensitization, irritation, systemic toxicity, material-mediated pyrogenicity, genotoxicity, implantation, hemocompatibility, carcinogenicity, reproductive/developmental toxicity, and degradation [17]. In the field of otorhinolaryngology, laryngectomy tubes are medical devices used to direct breathing through the upper airway for patients who have undergone laryngectomy procedures. These procedures involve removal of all or part of the larynx, often as treatment for laryngeal cancer. Unfortunately, supplies such as laryngectomy tubes are often not covered by insurance in the United States. Access to expensive supplies is further impaired as patients with laryngeal cancer are often of lower socioeconomic status [18]. Furthermore, tube sizes are standardized and may not comfortably fit each patient. The resulting discrepancies between an individual’s unique airway anatomy and a standardized laryngectomy tube can lead to discomfort and complications such as airway obstruction [19]. In addition, standard laryngectomy tubes undergo wear and generally need to be changed approximately every three months as biofilm formation can affect the structural integrity of the tube, as well as act as a source of infection [20, 21].

3D printing laryngectomy tubes have tremendous potential, offering a cost-effective solution that can be available for patients at the point of care. Furthermore, customization of these devices may prevent future costs of complication management due to improved fit and comfort.

However, since these devices are used in the upper airway, it is imperative to ensure good manufacturing practices and biocompatibility of these foreign materials in the human body. The development of bacterial biofilms poses a significant problem for these devices. It has been reported that biofilms are seen on more than 90% of tracheostomy tubes within 7 days of insertion [22]. In the context of laryngectomy tubes, presence of biofilms can lead to tracheitis, pneumonia, acute airway obstruction, and more [21]. *Staphylococcus aureus* (*S. aureus*) is a pathogen known to colonize implanted foreign bodies and artificial airways, leading to infection and *Staphylococcus* species account for two-thirds of infections associated with surgical implants. Such infection adds to the already heavy clinical and financial burden of device implantation [23–25]. Infection by *S. aureus* can be difficult to treat as the resulting biofilms are often not as responsive to antibiotics [26]. Further, infection due to implanted devices does not result in spontaneous healing, causing it to persist until the device is removed [27].

The presence of biofilms on 3D printed materials has primarily been studied in the context of development of anti-microbial polymers. However, there is a lack of knowledge about how biofilm growth compares between biocompatible 3D printed materials and standard traditionally manufactured implantable devices, especially with regards to laryngectomy tubes. The goal of this study is to assess the presence of *S. aureus* biofilms on a variety of 3D printed materials as compared to standard laryngectomy tubes in order to provide rationale for selection for optimal substrates for 3D printed versions.

## Materials and methods

### Specimen printing and preparation

Five biocompatible 3D printing materials including: two surgical guide resins (FormLabs, Somerville, MA and NextDent by 3D Systems, Soesterberg, the Netherlands), a photopolymer (VisiJet M3-X, 3D Systems, Rock Hill, SC), an elastomer (VisiJet ENT, 3D Systems, Rock Hill, SC), and a thermoplastic elastomer filament (DSM Arnitel, DSM, Herleen, Netherlands) were 3D printed and underwent post-processing using appropriate 3D printing technologies to form C-shaped discs (15 mm in height, 20 mm in diameter, and 3 mm in thickness), designed using Tinkercad (Autodesk, San Rafael, CA), exported as stereolithography (STL) files for printing, and printed using 3D printers specific to each material (Table 1). For comparison, Shiley™ laryngectomy tubes (size 6LGT) made from polyvinyl chloride (PVC) (MedTronic, Minneapolis, MN) were obtained and cut using a handsaw into C-shaped discs with similar dimensions as those of the printed discs.

### Sterilization

The 3D printed C-shaped discs were sterilized via autoclave dry cycle at 121 °C for 30 minutes, while slices of the laryngectomy tube were sterilized via low-temperature (37-44 °C) vaporized hydrogen peroxide gas plasma sterilization (STERRAD) for 28 minutes. All materials were sterilized according to manufacturer guidelines.

### Inoculation

**Fig. 1:**
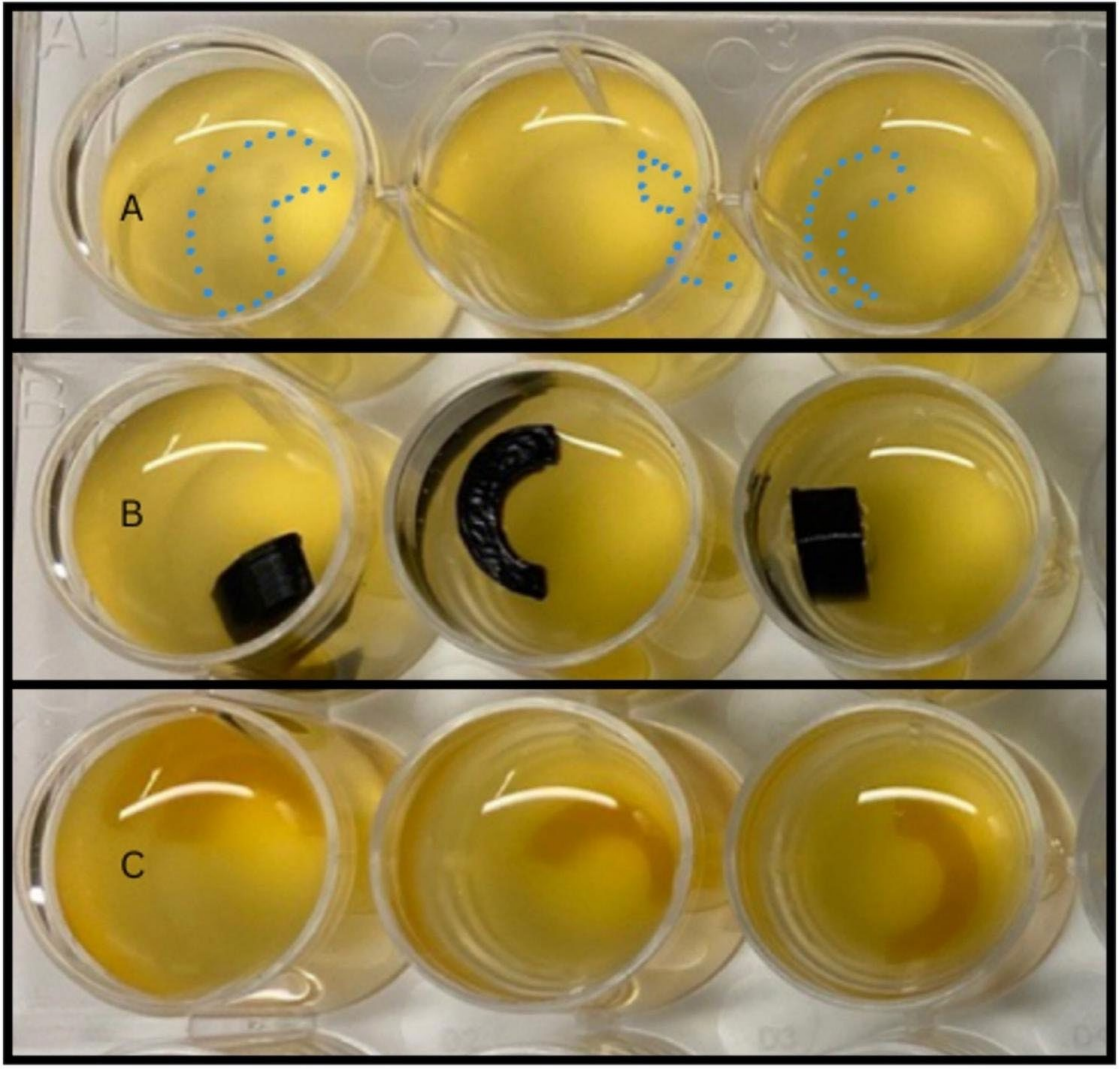
Setup of inoculation of three different types of 3D printed C-shaped discs (A: NextDent Surgical Guide Resin manually outlined due to poor visualization; B: DSM Arnitel; C: 3D Systems VisiJet ENT)

*S. aureus* cultures were created with 100 µL of bacteria in 10 mL of Tryptic Soy Broth (TSB) and were left in a 37 °C shaker for 3 hours. Using McFarland’s standard, the appropriate *S. aureus* dilution was determined and created in TSB. Discs of each material were placed into a 24-well plate and inoculated with 2 mL of the *S. aureus* cultures (Figure 1). The discs were incubated overnight at 37 °C. After incubation, all materials were removed from solution and washed in 2 mL phosphate-buffered saline (PBS) three times to remove planktonic bacteria. Next, they were placed in 15 mL falcon tubes with 10 mL PBS in which they were sonicated for 1 minute at 60% amplitude (Fisher Scientific, Hampton, New Hampshire) to detach biofilm bacteria. The discs were discarded, the remaining fluid diluted to 1/10^2^ and 1/10^3^ concentrations, 50 µL of the solution from each disc was plated onto Tryptic Soy Agar plates as technical triplicates, the plates were incubated overnight at 37 °C, and colony-forming units were manually counted the following day. One TSA plate was used per sample and three samples of each material were used in each experiment. Three separate C-shaped discs were printed and inoculated, and the fluid obtained after sonication from each specimen was plated on a single TSA plate.

### Replication

For the Shiley™ tube, FormLabs surgical guide resin, and 3D Systems VisiJet M3-X materials, there were eight samples of each material and testing was repeated over three experiments. The first experiment utilized two samples, each of which had three technical replicates, yielding six measurements. The second experiment used three samples of each material with two technical replicates for each, yielding six measurements. The last experiment had three samples of material with six replicates for each, yielding 18 measurements. Therefore, a total of 30 measurements were recorded for these three materials. The testing of NextDent surgical guide resin, 3D Systems VisiJet ENT, and DSM Arnitel materials was done in one experiment due to material limitations. Three samples of each material were used, and each sample was tested using 6 technical replicates, yielding 18 total measurements. Table 1 describes the 3D printing materials and methods used to create the 3D printed C-shaped discs.

**Table 1:** Description of 3D Printing Materials and Technologies used to create 3D printed C-shaped discs

Material	Slicing Software	Printing Technology	Printer Type	Number of Samples	Number of Measurements	Layer Thickness
FormLabs Surgical Guide Resin	Formlabs PreForm	Vat Photo-polymerization	FormLabs Form3	8	30	0.1 mm
NextDent Surgical Guide Resin	3D Systems 3D Sprint	Vat Photo-polymerization	3D Systems NextDent 5100	8	30	0.1 mm
3D Systems VisiJet M3-X	3D Systems 3D Sprint	Material Jetting	3D Systems ProJet MJP 2500 Plus	3	18	0.042 mm
3D Systems VisiJet ENT	3D Systems 3D Sprint	Material Jetting	3D Systems ProJet MJP 2500 Plus	3	18	0.042 mm
DSM Arnitel	Ultimaker Cura	Material Extrusion	Ultimaker S5	3	18	0.1 mm

### Statistical analysis

A Kruskal-Wallis analysis and Wilcoxon rank sum test were conducted to determine pairwise significance between the laryngectomy tube material and the 3D printed materials. All statistical analysis was performed in RStudio® Build 461 (RStudio PBC, Boston, MA).

## Results

Model discs were successfully printed using all 3D printing technologies and materials. Following sterilization and inoculation (Fig. 1), *S. aureus* demonstrated growth on all five 3D printed disc types and the control Shiley™ tube samples. Only the 1/10^3^ dilution concentrate ended up being used in the analysis as the 1/10^2^ dilution did not yield enough data for analysis.

**Fig. 2:**
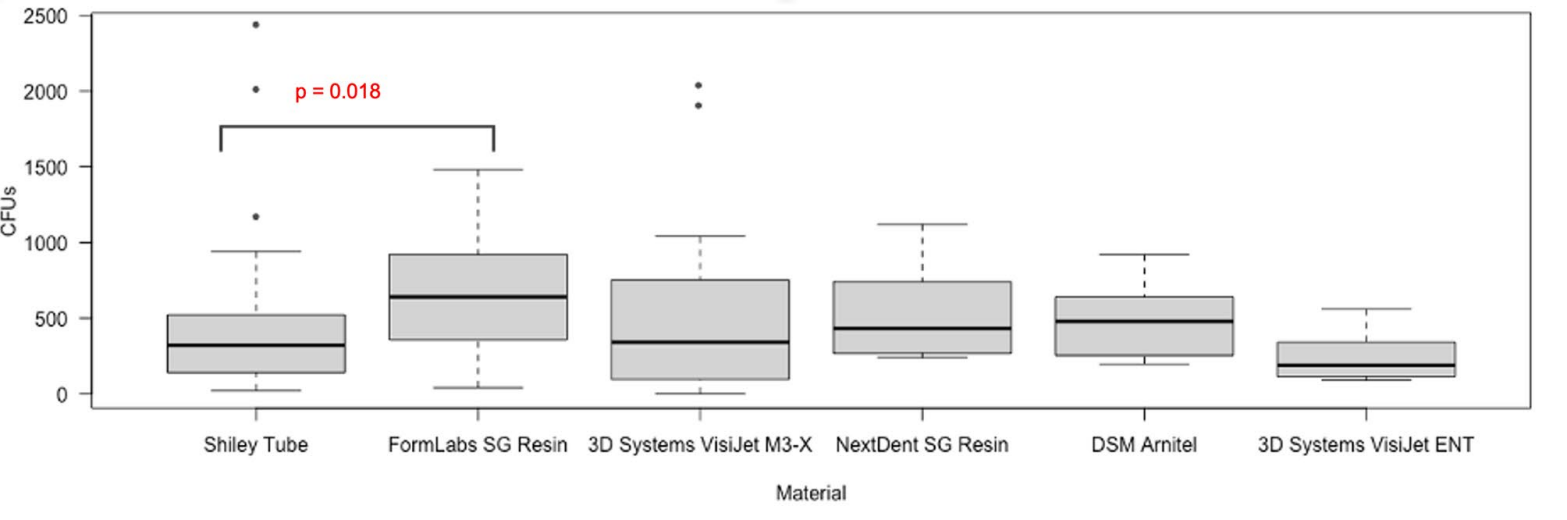
Comparison of biofilm growth on 3D Printed materials to Shiley control. The growth of each material was compared to that on the Shiley tube using a Wilcoxon rank sum test.

The Kruskal-Wallis test demonstrated a significant difference in colony growth between groups (H = 17.69, p < 0.01). The data was not normally distributed. Further, the post-hoc Wilcoxon rank sum test showed a significant difference between the Shiley™ tube and the FormLabs Surgical Guide Resin (p = 0.02) (Fig. 2). The least difference was seen between the Shiley™ tube and the 3D Systems VisiJet M3-X material (p = 0.89). Compared with the Shiley™ tube, the NextDent Surgical Guide Resin and DSM Arnitel both had p-values of 0.09 and the 3D Systems VisiJet ENT had a p-value of 0.17.

## Discussion

Medical devices that are marketed in the United States are subject to the regulatory controls in the Federal Food, Drug, and Cosmetic Act and its implementing regulations in Title 21 of the Code of Regulations [28]. Traditional manufacturers must first obtain premarket 510(k) clearance in order to manufacture a 3D printed device. In order to obtain clearance, the medical device industry routinely performs biocompatibility, toxicology, and sterilization validations for innovative medical products to ensure that they perform safely and effectively. Manufacturers often outsource biocompatibility and toxicology testing to external laboratories such as Nelson Labs though some perform these tests in-house. However, this is not commonly performed in hospitals as medical devices are typically bought from original equipment manufacturers. Furthermore, currently, hospitals performing 3D printing at the point of care are not subject to these regulatory controls since they typically utilize these devices in house and do not sell them.

It is important to note, however, that the Food and Drug Administration (FDA) is closely monitoring this landscape, has proposed several scenarios of 3D printing at the point of care, and will likely provide guidance for this in the future to ensure the safe and effective use of 3D printing in the hospital setting [28].

To our knowledge, there are limited studies evaluating biocompatibility of commonly available 3D printed materials. One study by Hall et al., focused on PLA polymers and evaluated growth of *Escherichia coli* and *Pseudomonas aeruginosa* in addition to *S. aureus.* They found biofilm growth was not only comparable on 3D printed polymers as compared to controls, but was even reduced [29]. These results differ from the results of our study, which showed an increase of growth for the FormLabs Surgical Guide Resin material.

This study was limited in several ways. The first limitation was the method of Shiley™ tube slicing used. They were cut using a handsaw; therefore, they may not be consistently the same size introducing possible variability in the surface area available for biofilm formation. Next, the number of materials tested along with the single geometry of the 3D printed part, the number of replicates, and the sample size were limited. These limitations were due to financial constraints of our laboratory as we were unable to procure additional materials and printers for testing. Furthermore, this study did not directly measure biofilm presence or volume. Future research could look to measure these variables directly via electron microscopy. Finally, this study focused solely on colonization by *S. aureus* and other microbes, including fungi, should also be examined.

In the future, creating 3D printed medical devices, such as laryngectomy tubes, may save time and cost for patients. This technology also offers a solution for patients who may not otherwise have access due to cost or supply chain barriers for manufactured items. With advancements in personalized 3D modeling based on medical imaging data, patient-specific, customized medical devices may lead to personalized items (such as form-fitted laryngectomy tubes), that are readily accessible at the point of care. For any healthcare delivery system endeavoring toward this, it is imperative to implement quality systems and check points to ensure the safe and effective use of these devices.

This study is a first of its kind – exploring a variety of potential materials for the production of point-of-care laryngectomy tubes. As this field grows, obviously a wide variety of materials should be tested in a robust fashion against an array of potential pathogenic microbes to arrive at optimal materials for 3D printed devices. The preliminary experiments performed herein suggest one such potential approach to identify safe materials for the creation of 3D printed laryngectomy tubes.

## Conclusion

In this study, *S. aureus* growth on five biocompatible 3D printed materials was quantified and compared to the growth seen on a conventionally manufactured medical device, the Shiley™ laryngectomy tube. The 3D printed materials tested had varying levels of biofilm colonization as compared to the Shiley™ laryngectomy tube. The FormLabs surgical guide resin demonstrated significantly higher colony formation. The DSM material also appeared to be more prone to colonization, though these results were not statistically significant (p > 0.05). Furthermore, neither the NextDent surgical guide resin, 3D Systems VisiJet M3-X, or 3D Systems VisiJet ENT materials demonstrated a significant difference in colonization as compared to the Shiley control. Consequently, all three of these materials show promise in the manufacturing of laryngectomy tubes at the point of care.

## Data Availability

All datasets are accessible through email to nicole.senderovich@einsteinmed.edu. No listed declarations are relevant to the content of this submission.
